# Relationship between malnutrition and possible sarcopenia in the AWGS 2019 consensus affecting mortality in hemodialysis patients: a prospective cohort study

**DOI:** 10.1186/s12882-021-02566-w

**Published:** 2021-11-12

**Authors:** Kenichi Kono, Yoshifumi Moriyama, Hiroki Yabe, Ayaka Hara, Takeki Ishida, Tetsuya Yamada, Yusuke Nishida

**Affiliations:** 1 Department of Physical Therapy, International University of Health and Welfare Graduate School, -3 Kozunomori, Narita, Chiba 286-8686 Japan; 2 Department of Rehabilitation, Kaikoukai Nagoya Kyoritsu Hospital, Nagoya, Aichi Japan; 3 Department of Physical Therapy, Seirei Christopher University. School of Rehabilitation, Hamamatsu, Shizuoka Japan; 4 Dialysis division at Shizuoka, Kaishokai, Shizuoka, Shizuoka Japan; 5 Dialysis division, Kaikoukai Healthcare Group, Nagoya, Aichi Japan

**Keywords:** Possible sarcopenia, Handgrip strength, GNRI, Mortality

## Abstract

**Background:**

The first objective of this study was to determine the relationship between muscle strength or physical performance and mortality, and the second objective was to show the relationship of Geriatric Nutritional Risk Index (GNRI) to muscle strength and physical performance decline.

**Methods:**

We examined handgrip, the 5-times chair stand test, and GNRI in 635 maintenance hemodialysis patients and followed up for 72 months. Predictors for all-cause death were examined using Kaplan-Meier analysis and Cox proportional analysis. The relationship between possible sarcopenia and nutritional disorder (GNRI) was constructed receiver operating characteristic (ROC) curve. We used the Youden index to determine the optimal cutoff points for GNRI.

**Results:**

The multivariate Cox proportional hazard analysis revealed that the GNRI did not show any significance, although handgrip (HR 3.61, 95% CI 1.70–7.68, *p* < 0.001) and the 5-times chair stand test (HR 1.71 95% CI 1.01–2.90, *p* = 0.045) were significant predictors for mortality. On the evaluation of possible sarcopenia by handgrip strength, the area under curve (AUC) on ROC curve analysis were 0.68 (95% CI 0.64–0.72), and 5-chair stand, the AUC on ROC were 0.55 (95% CI 0.51–0.60). The cut-off value for the GNRI discriminating those at possible sarcopenia by handgrip strength based on the Youden index was 91.5.

**Conclusions:**

Our study suggests that the handgrip strength test of the AWGS 2019 sarcopenia consensus was a simple and useful tool to predict mortality in chronic hemodialysis patients. Furthermore, GNRI assessment can be a useful tool for screening before assessing possible sarcopenia when it is difficult to perform SARC-F to all patients.

## Background

Protein-energy wasting syndrome (PEW), which causes adverse changes in nutrition and body composition, is highly prevalent in patients with chronic kidney disease, especially those undergoing dialysis, and is associated with high morbidity and mortality [1, 2]. The most important element of PEW in elderly patients with End-stage renal disease is the loss of muscle mass [3], which is common to the definition of sarcopenia. Sarcopenia is a progressive and generalized skeletal muscle disorder involving accelerated loss of muscle mass, low strength, and low physical performance [4]. Recently, the number of dialysis patients with elderly sarcopenia has been increasing due to aging, physical inactivity [5], and the presence of geriatric diseases and other comorbidities [6, 7]. This sarcopenia in dialysis patients is not a primary with no apparent cause other than aging, but secondary sarcopenia with several other factors as the cause. Therefore, it is clinically important to assess and detect malnutrition and sarcopenia for elderly dialysis patients.

The Asian Working Group for Sarcopenia (AWGS) [8] recommends case-finding to identify early signs of sarcopenia and introduces the concept of “possible sarcopenia” defined by low muscle strength with or without reduced physical performance in primary health care or community preventive service setting. However, the relationship between the reference value of these indexes for possible sarcopenia in AWGS2019 and outcomes such as mortality of dialysis patients has not been fully clarified.

Moreover, since few facilities have a physical therapist who can reliably evaluate the muscle strength and physical performance at an outpatient dialysis clinic, it would be useful to identify a screening index by existing clinical laboratory values that can replace indicators of possible sarcopenia. Recently, the geriatric nutritional risk index (GNRI) is used to assess serum albumin kinetics and physical condition and has been utilized as a nutritional assessment index in Japanese chronic kidney disease (CKD) patients [9, 10]. GNRI’s clinical usefulness for predicting mortality has also been reported [9, 11, 12]. If the GNRI can be used to extract sarcopenia cases, it will be effective in managing dialysis therapy by medical doctors, nurses, and clinical engineers.

Therefore, the first objective of this study was to determine the relationship between muscle strength or physical performance and mortality, and the second was to show the relationship of GNRI to muscle strength and physical performance decline.

## Methods

### Study population and design

This study included clinically stable Japanese outpatients in a multicenter hemodialysis clinic from April 2012 to April 2018. All patients who underwent hemodialysis treatment three times per week were included in the study. According to the Japanese Society for Dialysis Therapy data, this is the most common hemodialysis regimen in Japan. Patients were excluded from this study if they had been hospitalized within the previous 3 months, age < 18 years, dialysis vintage < 6 months, and refusal to participate. This study was approved by the Ethical Committee of the International University of Health and Welfare (Approval number. 17-Io-95).

### Demographic and clinical laboratory findings

Patients’ demographics, such as age, dialysis vintage, body mass index (BMI), primary kidney disease, and comorbid conditions, were investigated. Laboratory values of serum albumin, serum hemoglobin, C reactive protein, serum intact parathyroid hormone, standardized dialysis volume (Kt/V), and normalized protein catabolic rate (nPCR) were also collected.

### Assessment of nutritional status by the GNRI

The GNRI was calculated using the formula described elsewhere: *GNRI = [1.489 × serum albumin (g/L)] + [41.7 × (bodyweight/ideal bodyweight)]* [10, 13]. Ideal body weight was defined as having a BMI value of 22 kg/m^2^. Malnutrition was defined as a GNRI < 90, according to previous studies [9, 14].

### Measurement of muscle strength and physical performance

In determining possible secondary sarcopenia, muscle strength and physical performance were assessed with reference to the algorithms for the community in the AWGS 2019 consensus of the sarcopenia criteria. Muscle strength was assessed by handgrip strength to indicate skeletal muscle strength [8], and handgrip strength was measured using a Smedley-spring type dynamometer (101A HATS, Tokyo). The handgrip’s low muscle strength diagnostic cut-offs were < 28.0 kg for men and < 18.0 kg for women. Physical performance was assessed by the 5-times chair stand test [15]. The low physical performance diagnostic cut-off was ≧12 s [9]. All patients were measured for muscle strength and physical performance using the same method by a physical therapist at each facility on dialysis day before dialysis treatment.

### Outcomes

The primary study outcome was all-cause mortality. This outcome was assessed based on the death registry and medical records at the clinic.

### Statistics

Data are expressed as mean ± standard deviation (SD) or percentages, whenever appropriate. The relative risk of mortality for the muscle strength and physical performance as possible sarcopenia variables were estimated using Cox proportional hazard models with adjustment for age, sex, nutritional status, smoking status, dialysis efficiency, and comorbidities. Hazard ratios and their 95% confidence intervals (CI) were calculated using the estimated regression coefficients and their standard errors in Cox regression analysis. The survival curve was calculated by the Kaplan-Meier method. The *p*-value for comparison of the survival curve was determined by the log-rank test. The relationship between possible sarcopenia (handgrip strength, 5-times chair stand test) and nutritional disorder (GNRI) was constructed receiver operating characteristic (ROC) curves for outcomes using two variables. We used the Youden index to determine the optimal cut-off points for GNRI [16]. Youden index is used to measure the overall combined specificity and sensitivity of prognostic factor and is defined as the maximum vertical distance between the ROC curve and the diagonal of chance line and is calculated as maximum. The best Youden index is used to determine the best cut-off point of GNRI. *P*-value < 0.05 was considered statistically significant. Statistical analyses were performed using SPSS (version 25, IBM, Tokyo).

## Results

### Patient demographics, nutritional status, and probable sarcopenia index

A total of 635 Japanese patients were analyzed. Table 1 shows the patients’ demographics, nutritional status, and probable sarcopenia index.

**Table 1:** Baseline characteristics staratified by gender

Variable	Male (*n*=355)	Female (*n*=280)
Characteristicss		
Age (y)	70.5 (10.7)	68.6 (10.3)
Body mass index (kg/m^2^)	22.2 (3.38)	21.7 (4.26)
Smoking (%)	10.4	1.8
Dialysis vintage (m)	86.5(84.3)	87.6(85.3)
ESRD primary cause (%)		
Diabetes Mellitus	38.9	29.3
Nephrosclerosis	16.9	15.0
Nephritis	21.4	35.4
Other	22.8	20.4
Comorbid conditions (%)		
Diabetes Mellitus	33.4	27.6
Hypertension	63.3	53.3
Hyperlipemia	15.8	20.2
Laboratory and nutritional conditions		
Serum albumin (g/dL)	3.64 (0.72)	3.59 (0.29)
Serum hemoglobin (g/dL)	11.8 (0.96)	10.9 (0.95)
Serum C-reactive protein (mg/dL)	0.41 (0.82)	0.26 (0.58)
Serumn PTH intact (pg/mL)	159.8(96.2)	160.7(105.8)
Kt/V	1.48 (0.26)	1.76 (0.31)
Normalized protein catabolic rate	1.09 (0.22)	0.89 (0.19)
GNRI	92.4 (6.88)	91.5 (6.69)
GNRI<90 (%)	31.0	36.8
Physical Functioning		
Handgrip (kg)	25.5 (8.02)	17.7 (5.57)
Handgrip male<28kg, female<18kg (%)	62.5	52.9
5-times chair stand test (sec.)	11.8 (4.50)	11.4 (4.51)
5-times chair stand test ≧12seconds (%)	41.7	33.6

*ESRD* End stage renal disease, *PTH* Parathyroid hormone, *GNRI* Geriatric nutritional risk index

### Kaplan-Meier estimate and cox proportional hazards regression analysis of patients’ survival

**Fig. 1:**
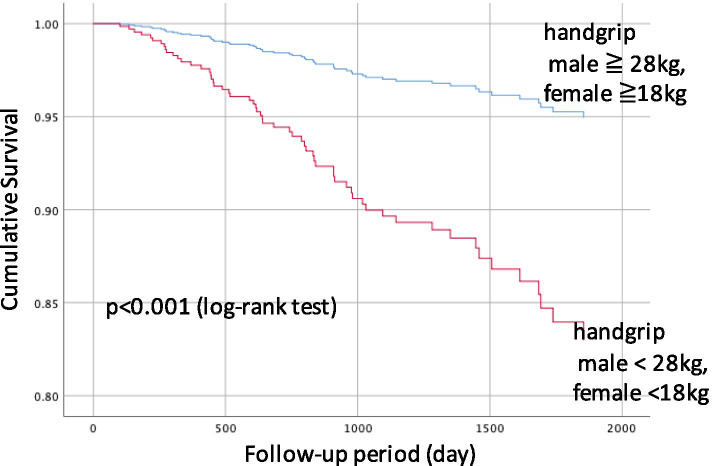
Handgrip and survival of hemodialysis patients. Patients with under cut-off value handgrip significantly lower survival rate during the follow-up period, compared with upper cutoff value (Kaplan-Meier analysis)

**Fig. 2:**
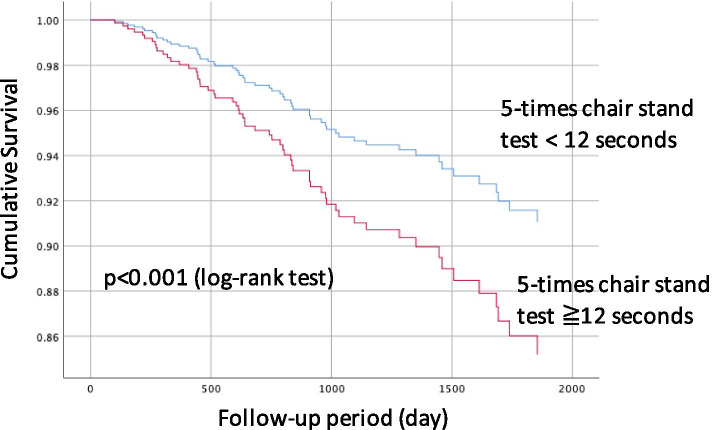
5-times chair stand test and survival of hemodialysis patients. Patients with 5-times chair stand test ≧12 s had a significantly lower survival rate during the follow-up period, compared with 5-times chair stand test <12 (Kaplan-Meier analysis)

The median follow-up of patients was 1003 days, during which 62 deaths (10%) were reported. Kaplan-Meier analysis revealed that patients with a handgrip and 5-times chair stand test below the possible sarcopenia cut-off had significantly lower survival rates than those with a higher function (log-rank test, *P* < 0.001, Figs. 1 and 2).

Univariate cox proportional hazards analysis for mortality showed that the handgrip and 5-times chair stand test were significant predictors for mortality (Table 2). Moreover, in the multivariate cox proportional hazards analysis, the handgrip (HR 3.61, 95% CI 1.70–7.68, *p* < 0.001) and the 5-times chair stand test (HR 1.71 95% CI 1.01–2.90, *p* = 0.045) were significant predictors for mortality (Table 2).

**Table 2:** Association between probable sarcopenia incices (handgrip, 5-times chair stand test) and mortality in cox proportional hazard regression models

	Handgrip male<28kg, female<18kg	5-time chair stand test ≧12 seconds
Univariate Model		
HR (95% CI)	4.86 (2.39 - 9.89)	1.92 (1.15 - 3.21)
*P*-value	.000	.013
Multivariate Model		
HR (95% CI)	3.61 (1.70 - 7.68)	1.71 (1.01 - 2.90)
*P*-value	.001	.045

Analyis were performing using Cox proportional hazards regression. Multivariate model included sex, age, normalized catabolic rate, Kt/V, diabetes mellitus, hypertension, hyperlipemia, smking

*HR* Hazard ratio, *CI* Confidence interval, *GNRI* Geriatirc nutritional risk index

### Relationship between nutritional status and possible sarcopenia indices

**Fig. 3:**
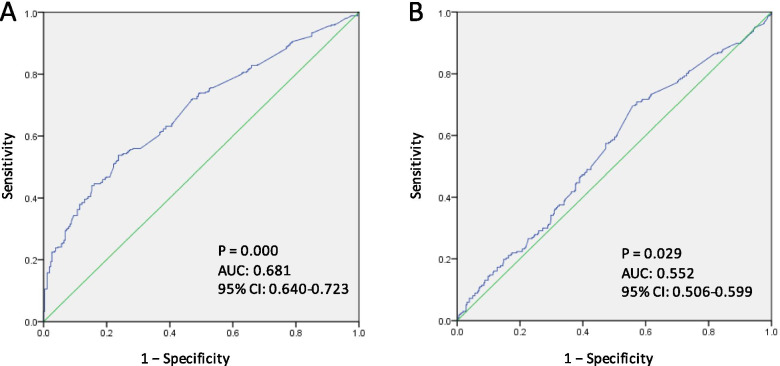
Receiver operating characteristics curves of GNRI for possible sarcopenia. Receiver operating characteristics curves of GNRI for possible sarcopenia by handgrip strength (**A**) and possible sarcopenia by 5-times chair stand test (**B**). In both (**A**) and (**B**), the AUC was significant, and (**A**) having a larger AUC. AUC, area under the curve; CI, confidence interval.

**Fig. 4:**
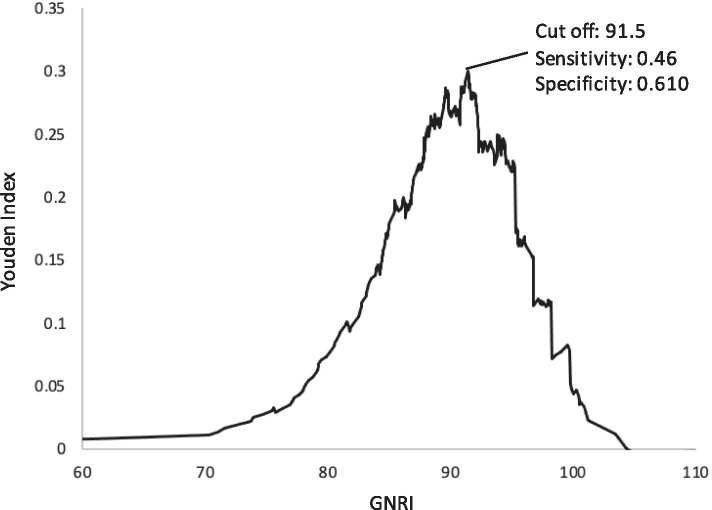
The cut-off value of GNRI detected by the Youden Index. GNRI, geriatric nutritional risk index

ROC curve analysis was performed for the GNRI (Fig. 3). On the evaluation of possible sarcopenia by handgrip strength, the area under curve (AUC) on ROC curve analysis were 0.68 (95% CI 0.64–0.72), and 5-chair stand, the AUC on ROC were 0.55 (95% CI 0.51–0.60). The cut-off value for the GNRI discriminating those at possible sarcopenia by handgrip strength based on the Youden index was 91.5 (Fig. 4).

## Discussion

In this prospective cohort and observational study, we clarified that handgrip and the 5-times chair stand test, indicated by the new sarcopenia consensus, the AWGS 2019, as a prognostic indicator for dialysis patients, are useful screening tools. In particular, handgrip strength was more than twice as high as the hazard risk compared to the 5-times chair stand test, which may be useful in terms of predicting mortality risk by assessing possible sarcopenia.

The relationship between reduced handgrip and mortality for dialysis patients has been previously reported [17] [18]. However, Vogt’s study differs from the conditions in our study in that the subjects’ were younger (as young as 50 years of age), and the cut-off value for handgrip reduction was 22.5 kg for males and 7 kg for females [17]. The finding of a significantly higher correlation between handgrip and mortality using the revised AWGS 2019 sarcopenia consensus appears to be a novel outcome. Furthermore, it is considered that in the results of our study, handgrip has been shown to correlate with systemic skeletal muscle function and malnutrition, which is linked to the results of this study suggest that handgrip was more strongly associated with mortality than the 5-times chair stand test [19–22].

The second finding of this study was that handgrip, a measure of possible sarcopenia, was significantly correlated with GNRI. The cut-off value for estimating handgrip strength loss for possible sarcopenia was 915. In a previous study of Japanese dialysis patients, the cut-off value for GNRI was reported to be 91–92 when the outcome setting was death [5, 6]. Our study of the cut-off value for probable sarcopenia was similar, suggesting that the risks of malnutrition, probable sarcopenia, and death are interrelated in the pathogenesis of PEW. Moreover, several studies on the usefulness of the GNRI in dialysis patients have shown that the GNRI has a higher sensitivity and specificity than other assessment tools for predicting mortality [23] [24]. Therefore, GNRI may be useful as a screening index based on medical record information as a substitute for SARC-F as described in the consensus [8] in estimating sarcopenia.

Another noteworthy aspect of this study is that the mortality rate during the observation period was 10%, which was a little lower than that of other country studies [11, 25], despite the large number of patients with low BMI. The reason for this may be that Japan’s universal health insurance system allows all dialysis patients to receive early treatment for complications, and it should be recognized that many patients may be thin because of differences in eating habits and not many PEW patients.

There were several limitations to our study. First, we have not been able to assess muscle mass, which is essential for sarcopenia diagnosis and can only refer to the relationship between the possible sarcopenia and mortality. Second, although we adjusted for comorbidities and other factors, this was an observational study, so interventional studies are needed to clarify the relationship between sarcopenia and death. Finally, although the GNRI cut-off value was presented as screening before possible sarcopenia assessment, the serum albumin level was not adjusted for diet or dialysis dose.

In conclusion, our study suggests that the handgrip strength test of the AWGS 2019 sarcopenia consensus (cut-off value, male< 28 kg, female< 18 kg) was the simple and useful tool to predict mortality in chronic hemodialysis patients. Furthermore, GNRI assessment can be a useful tool for screening before assessing possible sarcopenias, such as muscle strength and exercise performance, when it is difficult to perform SARC-F for all patients.

## Data Availability

The datasets used and/or analyzed during the current study are available from the corresponding author on reasonable request.

## Abbreviations

PEW: Protein-energy wasting syndrome
AWGS: Asian Working Group for Sarcopenia;
GNRI: Geriatric Nutritional Risk Index
SPPB: Short Physical Performance Battery
HR: Hazard ratio
95% CI: 95% confidence interval
BMI: Body mass index
Kt/V: Standardized dialysis volume;
nPCR: Normalized protein catabolic rate
SD: Standard deviation
CI: Confidence Interval
OR: Odds Ratio
